# Brain and Breast Cancer Cells with PTEN Loss of Function Reveal Enhanced Durotaxis and RHOB Dependent Amoeboid Migration Utilizing 3D Scaffolds and Aligned Microfiber Tracts

**DOI:** 10.3390/cancers13205144

**Published:** 2021-10-14

**Authors:** Annalena Wieland, Pamela L. Strissel, Hannah Schorle, Ezgi Bakirci, Dieter Janzen, Matthias W. Beckmann, Markus Eckstein, Paul D. Dalton, Reiner Strick

**Affiliations:** 1 Laboratory for Molecular Medicine, Comprehensive Cancer Center Erlangen-EMN (CCC ER-EMN), Department of Gynecology and Obstetrics, University Hospital Erlangen, Friedrich-Alexander University Erlangen-Nuernberg, Universitaetsstrasse 21-23, 91054 Erlangen, Germany; Annalena.Wieland@uk-erlangen.de (A.W.); Pamela.Strissel@uk-erlangen.de (P.L.S.); hannah.schorle@fau.de (H.S.); fk-direktion@uk-erlangen.de (M.W.B.); 2 Institute of Pathology, University Hospital Erlangen, Friedrich-Alexander-Universität Erlangen-Nürnberg, Krankenhausstrasse 8-10, 91054 Erlangen, Germany; Markus.Eckstein@uk-erlangen.de; 3 Adjunct Affiliation with Department of Radiation Oncology, University of Maryland School of Medicine, Baltimore, MD 21201, USA; 4 Department of Functional Materials in Medicine and Dentistry, Bavarian Polymer Institute, University Hospital Wuerzburg, Pleicherwall 2, 97070 Wuerzburg, Germany; ezgi.bakirci@fmz.uni-wuerzburg.de (E.B.); daltonlab@gmail.com (P.D.D.); 5 Institute for Clinical Neurobiology, University Hospital Wuerzburg, Versbacherstrasse 5, 97078 Wuerzburg, Germany; Janzen_D@ukw.de; 6 Knight Campus for Accelerating Scientific Impact, University of Oregon, 1505 Franklin Boulevard, Eugene, OR 97403-6231, USA

**Keywords:** 3D tumor model, 3D microfiber, amoeboid cell migration, brain cancer, breast cancer, PTEN, RHO, ROCK, durotaxis, topotaxis

## Abstract

**Simple Summary:**

Glioblastoma multiforme (GBM) and metastatic triple-negative breast cancer (TNBC) with *PTEN* mutations are associated with brain tumor spreading and poor patient outcomes. GBM, and possibly TNBC, migrate on axons and blood vessels to disseminate in the brain; however, the mechanism is unresolved. There is a need for new therapeutic targets to blunt brain tumor spreading. Using 3D aligned printed microfibers mimicking brain structures proved that RHOB, in addition to ROCK and PTEN signaling, were essential for GBM and TNBC 3D cell migration. GBM and TNBC cell lines with PTEN loss of function and high RHOB expression exhibited amoeboid morphology with increased durotaxis, binding and migration speed on 3D microfibers, in contrast to the PTEN wildtype. Depending on the *PTEN* genotype, RHO-ROCK-PTEN inhibitors or PTEN rescue significantly regulated these properties. Regarding GBM and brain metastasizing TNBC, we conclude that RHOB inhibitors could play a novel role for improved therapy response and patient outcome.

**Abstract:**

Background: Glioblastoma multiforme (GBM) and metastatic triple-negative breast cancer (TNBC) with *PTEN* mutations often lead to brain dissemination with poor patient outcome, thus new therapeutic targets are needed. To understand signaling, controlling the dynamics and mechanics of brain tumor cell migration, we implemented GBM and TNBC cell lines and designed 3D aligned microfibers and scaffolds mimicking brain structures. Methods: 3D microfibers and scaffolds were printed using melt electrowriting. GBM and TNBC cell lines with opposing PTEN genotypes were analyzed with RHO-ROCK-PTEN inhibitors and PTEN rescue using live-cell imaging. RNA-sequencing and qPCR of tumor cells in 3D with microfibers were performed, while scanning electron microscopy and confocal microscopy addressed cell morphology. Results: In contrast to the PTEN wildtype, GBM and TNBC cells with PTEN loss of function yielded enhanced durotaxis, topotaxis, adhesion, amoeboid migration on 3D microfibers and significant high RHOB expression. Functional studies concerning RHOB-ROCK-PTEN signaling confirmed the essential role for the above cellular processes. Conclusions: This study demonstrates a significant role of the *PTEN* genotype and RHOB expression for durotaxis, adhesion and migration dependent on 3D. GBM and TNBC cells with PTEN loss of function have an affinity for stiff brain structures promoting metastasis. 3D microfibers represent an important tool to model brain metastasizing tumor cells, where RHO-inhibitors could play an essential role for improved therapy.

## 1. Introduction

Cell migration and invasion are hallmarks of development and cancer [1]. For the ability of cells to migrate, dynamic and spatially regulated changes of the cytoskeleton, cell adhesion and cell interactions with the extracellular matrix (ECM) must occur [2]. Depending upon the cell type, two main migratory phenotypes can be differentiated: (1) single cells, which are amoeboid or mesenchymal, and (2) multicellular, or so-called collective cell migration [3]. Cell-cell as well as cell-ECM interactions are crucial for a cell to instantly respond to the environment and further propagate the signals that control cell shape and motility [4]. Specific tissue types harbor different ratios of ECM components, such as collagen, laminin, fibronectin and hyaluronic acid [5]. Especially in the brain, higher amounts of laminin and hyaluronic acid are found, where they contribute to normal brain function and tumor stiffness regulating tumor cell motility [6,7]. Although laminin is not a component of axons, it is embedded in an ECM enriched with laminin. Collagen is not an ECM component in normal brain but is found in the basement membrane of blood vessels. Primary GBM and human GBM xenografted mice showed increased levels of collagen in tumors from tissue microarrays using second-harmonic generation microscopy [8]. These authors found a significant association of disorganized collagen filaments in GBM with a poorer patient survival. Several different laminin proteins compose an inner blood vessel basement membrane and an outer one with capillaries. Interestingly, within the specialized perivascular spaces containing blood vessels surrounded by cerebral spinal fluid, laminin encases pericytes on capillaries and also locates at the outer surface [9]. Functionally, brain tumor cells sense ECM structures as migratory cues, where they become polarized by reorganizing the actin cytoskeleton to facilitate both a protrusive leading and a contractile trailing edge [10]. Cellular protrusions with organized actin filaments include lamellipodia, filopodia, invadopodia and blebs, which are regulated by various cytoskeletal and signaling pathways [11].

Glioblastoma multiforme (GBM) arise from glial cells of the central nervous system and represent the most aggressive type of human brain tumors [12,13,14]. Due to the diffuse infiltration of GBM throughout the brain, the inability to achieve complete surgical tumor resection leads to poor patient prognosis with a mean survival of about 15 months [14]. Although circulating GBM cells have been detected within the blood in 20% of patients at primary diagnosis, extracranial metastases of GBM are rarely found, most likely due to short patient survival [12]. Therefore, it is crucial to obtain a better understanding of GBM migration at a cellular and molecular level.

It is known that GBM cells are attracted to white matter tracts composed of myelinated axons organized into bundles, but interestingly also on blood vessels within the perivascular spaces where GBM cells migrate and metastasize surrounding brain regions [13,15,16]. One explanation for GBM cell attraction could relate to the high mechanical rigidity of axons and blood vessels. This type of physical attraction towards a more rigid environment where cells migrate to and “home in” is defined as durotaxis, or mechanical substrate compliance [7,17]. It has been shown that integrin-based focal adhesions are stably or dynamically tracking/sensing the stiffness critical for durotaxis and migration speed [18]. Other environmental cues influencing cell migration are as follows [18]: chemotaxis, where cells migrate towards diffusible chemical gradients; haptotaxis representing cell movement towards chemical cues on a surface, such as ECM proteins and, lastly, contact guidance, also called topotaxis or ratchetaxis, where cells use ultrastructures as guiding tracks for migration. Topotaxis was described as a mechanism where a single cell membrane and the cytoskeleton conform to the topography of the cell adhesion substrate. Using melanoma cells, it was shown that this adaptation to the topography was dependent mainly on genetic and signaling differences involving the PI(3)K and ROCK-dependent pathways [18]. In addition, it was shown that nanostructures, which translate topographical signals to cells, influenced directional migration [19,20].

Distant metastasis of primary tumors are known, especially triple negative breast cancer (TNBC), which has a brain metastasis incidence rate of 46% [21,22]. TNBC is a highly malignant and fast progressing subtype of breast cancer that is diagnostically negative for the expression of the estrogen and progesterone receptors with no overexpression of the human epidermal growth factor 2 receptor. Research is ongoing about how metastasis of circulating TNBC cells to the brain occurs, but how these cells enter and disseminate throughout the brain and if they share similar cellular activities to GBM cells is still unanswered.

At the molecular level, a key player involved in regulating cellular migration and invasion is the Phosphatase and Tensin Homolog (PTEN) protein. PTEN is a known tumor-suppressor antagonizing the oncogenic phosphoinositide 3-kinase (PI3K) pathway, where it dephosphorylates the metabolite phosphatidylinositol-3,4,5-triphosphate (PIP3) to phosphatidylinositol-4,5-bisphosphate, inhibiting downstream signaling. The mechanism of PTEN in motile cells was shown due to its cellular localization and dynamics with PIP3. PIP3 is located at the leading edge of migrating cells, stimulating polymerization of actin. PTEN suppresses cell motility by dephosphorylating PIP3 resulting in lower actin polymerization. A cross-inhibitory role of PIP3 and PTEN was proposed where a rise of PIP3 levels led to the dissociation of PTEN from the membrane, and this cycling process resulted in PIP3 waves [21,22]. PTEN loss of function results in constitutively active PI3K signaling and induces proliferation, migration and cell survival [23,24]. PTEN mutations are one of the most common genetic alterations in GBM and are directly associated with malignant transformation and metastasis, but also noted in up to 40% of primary breast cancers associated with therapeutic resistance and a shorter patient overall survival [25,26,27,28,29]. Interestingly, PI3K activation or loss of PTEN was found in 77% of brain metastases or 25–71% of breast tumors metastasized to the brain, respectively [30,31,32].

Other essential factors for cell migration are the ras homolog family member (RHO) GTPases, which include 20 mammalian genes and play a more determining role regulating actin polymerization, depolymerization and activity of actin-associated myosins during migration [33]. RHO GTPAses cycle between an inactive (GDP-bound) and active (GTP-bound) form, regulated by the activity of Rho-specific guanine nucleotide exchange factors (exchange of GDP with GTP) and GTPase-activating proteins that catalyze GTP-hydrolysis. In addition, most RHO GTPAses, such as RHOA, also translocate between cytosol (inactive) and membrane (active) forms, due to modification by isoprenyl lipids [33]. PTEN is required for directional movement of cells via different cellular localizations and activation through RHO GTPase and Rho associated coiled-coil containing protein kinase (ROCK) signaling [34]. Besides the main RHO GTPase RHOA, RHOB plays an essential role for actin cytoskeleton and especially for membrane blebbing and amoeboid migration [35].

Biomedical materials developed for use in tissue engineering are essential for modeling 3D tumor growth, migration and invasion [36,37]. A relatively new 3D printing technology, melt electrowriting, has been used to fabricate complex multiscaled architectural biomaterial structures [38,39] from microfibers [40], also used for clinical purposes. Melt electrowriting processes polymers, which can be implemented within a biological context for 3D cell cultures recapitulating aspects of the in vivo environment, are novel tools for 3D tumor cell modeling [41,42,43].

In this investigation two different 3D melt electrowriting poly-(ε-caprolactone) (PCL) structural designs were used as topographical guides to mimic axons and blood vessels in the brain to assess if GBM and TNBC cells have similar cellular activities. RNA sequencing (RNA-seq) was used to shed light on the gene pathways regulating adhesion, migration and cell morphology. Our main findings demonstrate that GBM and TNBC types have enhanced durotaxis and topotaxis and increased migration influenced by the PCL melt electrowriting fibers. We show new findings that enhancement of the above cellular activities stems from mutated *PTEN* variants with loss of function and de-regulated RHOB signaling, which is dependent on a 3D environment. This study validates a significant role for the *PTEN* loss of function genotype for these cellular processes and explains why these tumor cells have a greater affinity for stiffer structures, such as axons and blood vessels promoting dissemination throughout the brain.

## 2. Materials and Methods

### 2.1. Cell Lines and Cell Culture

All cell lines used in this investigation were purchased from the American Type Culture Collection (ATCC, Rockville MD, USA) and tested regularly for mycoplasma infection according to the manufacturer’s instructions (Minerva Biolabs, Berlin, Germany). GBM cell lines U87 MG (U87) (ATCC^®^ HTB 14™) and LN18 (ATCC^®^ CRL-2610™) and the triple negative breast cancer (TNBC) cell lines MDA-MB-231 (ATCC^®^ HTB26™) and MDA-MB-468 (ATCC^®^ HTB132™) were grown and maintained as described below. U87 cells were cultured in Minimum Essential Medium (MEM); LN18 and MDA-MB-231were cultured in Dulbecco’s modified Eagle’s medium (DMEM) and MDA-MB-468 in Nutrient Mixture F-12 (DMEM/F12). All media were supplemented with 10% fetal bovine serum (FBS), 2 mM L-Glutamine, 10 mM HEPES Buffer and 1 x nonessential amino acids (all media, FBS, and supplements from Gibco™ Thermo Fisher Scientific, Waltham, MA, USA). Cells were maintained in 75 cm tissue culture flasks in a humid atmosphere with 5% CO_2_ at 37 °C. Cells were passaged at 70–80% confluency using 0.0625% Trypsin. The U87 cell line has a *PTEN* gene splice donor mutation of exon three resulting in an in-frame exon three deletion and PTEN loss of function, whereas LN18 is *PTEN* wild type (wt) [44]. The TNBC MDA-MB-468 cell line has a *PTEN* gene splice donor mutation of exon four resulting in an in-frame deletion of exon four and PTEN loss of function [44,45]. The MDA-MB-231 cell line is *PTEN* wt. For simplicity, both U87 and MDA-MB-468 cell lines are referred to as PTEN loss of function, whereas LN18 and MDA-MB-231 as PTEN wt.

### 2.2. Melt Electrowriting Printing and Glass Slide Treatment

A custom-built melt electrowriting printer was used to fabricate box-pore scaffolds as well as aligned and stacked microfiber tracts as previously described [38,46]. Briefly, melt electrowriting was performed using medical-grade PCL (PURASORB PC 12, Lot#1712002224, 05/2018, Corbion Inc, Amsterdam, Netherlands) at 21 ± 3 °C and a humidity of 40 ± 5%. Scaffolds were printed at 85 °C, 3 bar, 25 G nozzle, 4 mm collector distance and 6 kV voltage applied. A 48 mm × 96 mm rectangular mesh was direct-written and cut to 9 mm disks with an infrared laser. Aligned microfiber tracts were printed at 85 °C; 0.5 bar of air pressure; 22 G nozzle and 6.5 kV voltage applied across a 3.85 mm collector distance. The interfiber distance was set at 200 µm with 10 layers in G-code for both scaffolds. Scaffolds and aligned microfibers were printed onto a metal surface or glass coverslips, respectively, where the glass surface was previously coated with NCO-terminated, star-shaped poly(ethylene oxide-*stat*-propylene oxide) (sP(EO-*stat*-PO) (provided by DWI Leibnitz Institute for Interactive Materials, Aachen, Germany) to facilitate scaffold adherence but decrease surface protein adsorption. Prior to sP(EO-*stat*-PO) coating, coverslips were washed with acetone, water, and isopropanol and dried using an air pressure gun, treated with 100% oxygen plasma in a plasma generator (Pico low-pressure plasma system, Diener Electronic GmbH, Ebhausen, Germany) then incubated in a desiccator with 3-aminopropyl-trimethoxysilane for surface activation. Coverslips were then homogenously rotationally spun and coated at 2500 rpm for 40 s with 10 mg mL^−1^ NCO-sP(EO-*stat*-PO) in 10% (*v*/*v*) tetrahydrofuran (Merck, Darmstadt, Germany)–MilliQ water. Coated coverslips were ready for melt electrowriting printing within 24 h.

### 2.3. Melt Electrowriting Scaffold Functionalization

3D box-pore scaffolds were cut into 9 mm discs and placed into a 15.6 mm culture dish (24-well plate) (Waltham, Massachusetts, MA, USA) and 3D-aligned microfiber tracts were placed into a 34.8 mm culture dish (6-well plate) (Corning, New York, NY, USA) then sterilized with UV-light for 30 min, incubated with 1 M NaOH for 10 min and finally washed five times with 1x PBS. For functionalization, scaffolds or aligned microfiber tracts were incubated with 10 µg mL^−1^ laminin-111 (BioLamina, Sundbyberg, Sweden) at 4 °C overnight.

### 2.4. U87 Cells Stably Express the Farnesylated-tdTomato Fluorescent Protein

A stable transfected U87 cell line was established expressing the farnesylated-tdTomato fluorescent protein in order to track the 3D movement of cells within Matrigel with or without scaffolds (for cell network formation assay see below). For cloning of the td-farnesyl-vector, the cDNA of the farnesylated-tdTomato fluorescent protein was reversed transcribed from the tdTomato-farnesyl-5 gene (Addgene, Watertown, MA, USA) implementing primers containing a Bam HI and NotI restriction enzyme sites in order to replace the AcGFP cDNA in the pLVX-AcGFP-N1 vector (Clontech, Mountain View, CA, USA). Following ligation into the pLVX-AcGFP-N1 vector, bacterial transformation, purified plasmid DNA was then expanded using the NucleoBond Xtra Maxi Kit (Macherey-Nagel, Düren, Germany) and verified for the correct tdTomato-farnesyl-5 gene sequence of the construct (Eurofins Genomics Germany GmbH, Ebersberg, Germany). The pLVX-tdTomato-farnesyl-5-N1 vector was then cotransfected with the packaging plasmid psPAX.2 (Addgene, Watertown, MA, USA) and the envelope vector VSV-G (Addgene, Watertown, MA, USA) into LentiX 293 T cells (Takara Bio Inc., Kusatsu, Shiga, Japan) using the Lipofectamine 2000 reagent (Thermo Fisher Scientific, Waltham, MA, USA). Forty-eight hours after transfection, the lentivirus-containing supernatant was harvested and briefly centrifuged before being concentrated with the LentiX concentrator (Takara Bio Inc., Kusatsu, Shiga, Japan). For subsequent reverse transduction of U87 cells, the concentrated virus (107 infectious units mL^−1^) was dispensed at 100,000 cells per 9.6 cm^2^. Successfully transduced U87 cells were further selected with 3 µm mL^−1^ Puromycin (Thermo Fisher, Waltham, MA, USA).

### 2.5. Cell Network Loop and Branch Formation Quantification

In order to perform the cell network formation assay, each cell suspension contained 50,000 U87 farnesylated-tdTomato cells and Matrigel (Corning, Corning, New York, NY, USA) at a final concentration of 4.5 mg mL^−1^ in a final volume of 150 µL, and then was pipetted onto a laminin-coated scaffold in a 15.6 mm culture dish. After 30 min incubation at 37 °C, 100 µL culture medium was added. Laminin-coated scaffolds embedded in Matrigel were imaged every 10 min at 568 nm for a total of 20 h using an inverted microscope (Olympus IX83, cellSens Software V1.16, Olympus Corporation, Tokyo, Japan) using a live cell imaging chamber at a temperature of 37 °C and at 5% CO_2_. All cell culture experiments were maintained in this live cell imaging chamber during imaging. Six experiments were performed for U87 cells with scaffolds and eight experiments with U87 cells in Matrigel alone. The AVI-videos were converted into TIF-files using a Python script. The cell network formation assay was analyzed using the online Tool WimTube (Wimasis, Onimagin Technologies SCA, Cordoba, Spain). We previously implemented the WimTube program to analyze primary breast adipose stem cells, which form and resemble endothelial capillary like structures with Matrigel [43]. The WimTube-Tool software performs quantification of tube-like formations from microscopic images. It is an automated process that recognizes cellular networks, which form either closed tube-like or loop network structures (counted independently with numbers indicated in yellow), but also quantifies branch-like structures (marked in white), which are not closed in formation. In this present study, the following structures were quantified using WimTube for the cell network formation assay with U87 cells: (1) the total number of cell loop structures and (2) the total number of branch-like structures. 

### 2.6. Cell Migration Assessment Following Treatment with PTEN, RHO and ROCK Inhibitors or Rescuing PTEN Function with Live Cell Imaging

Live cell imaging was performed using an inverted microscope and phase contrast (Olympus IX83, cellSens Software V1.16, Olympus Corporation, Tokyo, Japan). All cell culture experiments were maintained in this live cell imaging chamber during imaging. For each migration experiment, 20,000 cells of a respective cell line were initially seeded onto laminin-coated aligned microfiber tracts and, after a 15 min incubation at 37 °C, 100 µL of culture medium was added. Aligned microfibers were imaged every 10 min for up to 15 h in an enclosed chamber with constant equilibration at 37 °C and 5% CO2. The AVI-videos were converted into TIF-files using a Python script. To inhibit the PTEN wt protein, LN18 cells (300,000 per 9.6 cm^2^) were treated with the PTEN inhibitor SF-1670 (Sellekchem, Houston, TX, USA) at a concentration of 3 µM starting 48 h prior to migration and live cell imaging (*n* = 7 experiments). To rescue U87 cells with a PTEN loss of function, we cloned the human *PTEN* wt gene under control of CMV in the overexpressing pRK5 vector. *PTEN* cDNA was reversed transcribed from human whole blood RNA using primers containing an EcoR1 and EcoR1/Sal1 sites. The PTEN PCR product (1230 bp) was ligated into the pRK5 Vector (pRK5-PTEN) and confirmed by DNA sequencing. U87 cells with a PTEN loss of function were transfected with 3 µg pRK5-PTEN vector using JetPEI^®^ according to the manufacturer’s instructions (VWR, Radnor, Pennsylvania, PA, USA). The U87 PTEN transfected cells were assayed for migration on laminin-coated aligned microfibers using live cell imaging after 48 h (*n* = 4 experiments for control; *n* = 3 experiments for transfection). To inhibit the RHO-associated, coiled-coil containing protein kinase (ROCK), we treated U87 cells (350,000 per 9.6 cm^2^) with the ROCK inhibitor Y-27632 (Stemcell Technologies, Vancouver, Canada) at a concentration of 5 µM, starting 24 h prior to the migration assay using live cell imaging (*n* = 7 control and *n* = 9 ROCK inhibitor experiments). For RHO inhibition of respective cell lines (350,000 per 9.6 cm^2^), 3 µg/mL of the RHO Inhibitor (C3 Trans based) (Biozol, Eching, Germany) was added to culture media containing 5% FCS, starting 6 h prior to the migration assay live-cell imaging. Cells were then incubated with laminin-coated aligned microfiber tracts and imaged every 10 min for a total of 20 h (U87 PTEN loss of function: *n* = 4 experiments, MDA-MB-468 PTEN loss of function: *n* = 3 experiments). AVI-videos were converted into TIF files using a Python script. The migration speed in µm/h was calculated using the manual tracking tool of ImageJ/Fiji.

### 2.7. Cell Viability

Cell viability after treating cell lines with the RHO inhibitor was assessed using a Live/Dead assay. In brief, cells were incubated for 30 min at 21 °C for 30 min with 2 × 10^−6^ M Calcein-AM (green = living cells; Thermo Fisher Scientific, Waltham, MA, USA) and 2 × 10^−6^ M ethidium homodimer I (red = dead cells, Sigma-Aldrich, St. Louis, MO, USA) in 1x PBS.

### 2.8. Immunocytochemistry and F-Actin Staining

U87 cells (50,000 cells grown in 4.5 mg mL^−1^ Matrigel (Corning, NY, USA) with scaffolds were stained for ß-Tubulin and microtubule-associated protein 2 (MAP2) to assess 3D cell growth and network formation at day 1, day 3 and day 6. All steps were performed at 21 °C. Cells were fixed for 15 min with 2% paraformaldehyde (PFA), washed with 1x PBS, and blocked/permeabilized for 3 min with 5% goat serum and 0.2% Triton X-100 in 1x PBS. Cells were incubated with the primary antibodies Anti-ß-Tubulin (1: 500, Abcam, Cambridge, UK) and Anti-MAP2 for 2 h (1: 500, Sigma Aldrich, St. Louis, MI, USA). Following washing, cells were incubated for 45 min with secondary Dylight-488 Alexa and Dylight-594 (both 1:200 dilutions; Sigma Aldrich, St. Louis, MI, USA). For F-actin staining, Actin Green 488 ready probes were used (Thermo Fisher Scientific, Waltham, MA, USA). To stain cell nuclei DRAQ5 (BioStatus, Shepshed, UK) or Hoechst 33342 (Thermo Fisher Scientific, Waltham, MA, USA) were used according to the manufacturer’s instructions. The computer software Imaris was used for 3D reconstructions (Oxford Instruments, Abingdon, UK). Cells were either incubated with Actin Red 555 Ready Probes Reagent (2 µg/mL) (Rhodamine phalloidin) or Actin Green 488 ready probes (both from Thermo Fisher Scientific, Waltham, MA, USA) specific for F-actin, and DRAQ5 (Biostatus Ltd., Shepshed, UK) for cell nuclei according to the manufacturer’s instructions.

### 2.9. Confocal and Scanning Electron Microscopy

Cells were imaged using a confocal laser scanning microscope (Leica, SP5X, 20 x dip-in water-immersion objective; numerical aperture (NA) 1.0) with laser power set to 1.2 mW to avoid photobleaching. F-Actin Alexa 488 was detected with an Argon laser at 488 nm; F-Actin Red 555 (Tetramethylrhodamine (TRITC)) with an Argon laser at 540 nm, and DRAQ5 with a He-Ne laser at 633 nm. Hoechst 33342 was detected with a UV laser at 350 nm. Images of cells with scaffolds represent an overlay of 35–40 z-stack sections. Confocal images of cells on aligned microfibers represent an overlay ranging from 18–21 z-stack sections (each step 0.99 μm equaling 17.82–20.79 μm with a line average of 3).

For scanning electron microscopy, a Zeiss Crossbeam 340 scanning electron microscope (Carl Zeiss Microscopy GmbH, Oberkochen, Germany) was used. Aligned microfibers were washed with PBS and incubated in 6% glutaraldehyde (Sigma-Aldrich, Schnelldorf, Germany) for 15 min. After a washing step in ice-cold PBS, the samples were dehydrated by increasing concentrations of ethanol from 50 to 100%. The samples were incubated in hexamethyldisilazane (Sigma-Aldrich, Schnelldorf, Germany) for 15 min and dried overnight. All samples were sputter-coated with a 4 nm layer of platinum with a Leica EM ACE600 (Leica Microsystems, Wetzlar, Germany) before imaging.

### 2.10. RNA Extraction, qPCR and RNA-Sequencing

For RNA-extraction, 50,000 cells of representative cell lines were grown in Matrigel in the presence or absence of scaffolds compared with cells grown on 2D culture dishes alone or coated with Matrigel at 0.1 mg mL^–1^. RNA was extracted at day 3 using TriFast^®^ PeqGOLD (PEQLAB Biotechnologie, Erlangen, Germany) according to the manufacturer’s instructions. All samples were incubated with DNase I to further fractionate the RNA and then precipitated in the presence of glycogen (both from Sigma-Aldrich, St. Louis, MI, USA). Human brain total RNA from a single healthy normal donor from freshly harvested tissue was purchased (Amsbio, catalog number HR-201). All RNAs were stored at −80° C. RNA was reversed-transcribed into cDNA using the High-Capacity cDNA Reverse Transcription Kit (Thermo Fisher Scientific, Waltham, MA, USA). For quantitative real-time PCR, SYBR Select Master Mix (Life Technologies, 4472919) and MicroAMPFast 96-well Reaction-plates (Applied Biosystems, Forster City, CA, USA) were used with a StepOne^TM^ Real-Time PCR System (Thermo Fisher Scientific, Waltham, MA, USA). All kits were used accordingly to the manufacturer’s instructions. Primer Sequences: RPL13-TF (5′-CTGCTGAAGAACTGAAACTGGC-3′), RPL13-BR (5′-CTCTTCCTCAGTGATGACTGGA-3′), RHOA-TF (5′-TTCCCAAGAAACTGG-3′), RHOA-BR (5′-CATACACCTCTGGGA-3′), RHOB-TF (5′-CATCCAAGCCTACGA-3′), RHOB-BR (5′-CAGTTGATGCAGCCGTTCTG-3′), MAP2-TF (5′ AAGAGAATGGGATCAACGGAGAG), MAP2-BR (5′ TTGTTCACCTTTCAGGACTGCT).

For RNA-sequencing (RNA-seq) 200 ng poly-A purified mRNA was isolated from U87 cells grown in 2D and in 3D with Matrigel and scaffolds from day 3 (*n* = 3 independent experiments were pooled). RNA-seq was performed by Eurofins Genomics Germany (Ebersberg, Germany). Paired-end read RNA-seq (2 × 150 bp) was executed via Illumina with 31,779,803 clean reads for U87 in 2D and 33,331,487 for U87 in 3D and scaffolds. Transcriptome analyses were performed by mapping and quantification of transcripts against a reference genome and a pairwise comparison of expression determining significant fold changes.

### 2.11. Western Blot

Cell lysates were loaded onto Mini-Protean TGX Precast protein gels (BioRad, Hercules, CA, USA) and run at 100 V for 1 h. Gels were semi-dry transferred onto PVDF membranes. The membrane was incubated in 1 x PBS + 0.1% Tween-20 buffer (1x PBST) with 3% milk powder (blocking buffer) for 1 h at room temperature. A PTEN antibody (Cell Signaling, Danvers, Massachusetts, MA, USA) and a GAPDH antibody (Santa Cruz Biotechnologies, Dallas, Texas, TX, USA) were diluted 1: 750 in blocking buffer and incubated overnight at 4° C. Washes were performed with PBST before the addition of goat-anti-rabbit-HRP (Cell Signaling, Danvers, MA, USA) (1:500) for 1 h at room temperature. For protein detection, the membrane was incubated for 3 min in HRP juice (PJK GmbH, Kleinbittersdorf, Germany) and visualized using the Amersham^TM^ Imager 600 (GE Lifesciences, Little Chalfont, UK). Original Western blot images can be found in Figures S4 and S5.

### 2.12. Statistical Analysis and Data Presentation

Statistical analyses were performed using GraphPad Prism 8 for Windows (GraphPad Software Inc., San Diego, CA, USA) with nonparametric statistical tests (Mann-Whitney-Test). Breast and glioblastoma pan cancer atlas, and The Cancer Genome Atlas (TCGA) data sets were accessed via cbioportal [47]. Categorized mutational data for *PTEN* (PTEN mutation = pathogenic and likely pathogenic mutations; *PTEN* splice variants and wt) and *RHOB/RHOA* gene expression were downloaded and correlated with each other. For statistical testing nonparametric Mann-Whitney tests were used.

## 3. Results

### 3.1. Design and Fabrication of 3D Scaffolds and Aligned Microfibers for Cell Functional Studies

**Figure 1:**
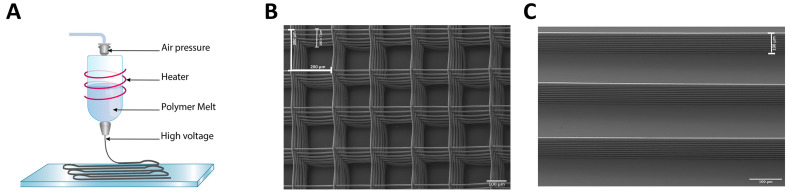
Fabrication and design of 3D scaffolds and aligned microfibers have similar properties. (**A**) Schematic representation of melt electrowriting. Medical grade PCL melt was extruded through an electrified nozzle into a jet that solidified upon reaching the collector. Repeated deposition enabled the fabrication of a 9 mm diameter scaffold or an 8 mm long array of aligned microfibers. (**B**) Scanning electron microscopy image of a scaffold consisting of 10 stacked aligned fibers along the length and 20 stacked overlapping aligned fibers at each corner, where each fiber has a diameter of 10 µm. The interfiber bundle distance is 200 µm (indicated as a white bar) with an overall box form scaffold structure with the total height of the fiber walls ~ 140 ± 7 µm as indicated (in white). (**C**) Scanning electron microscopy image of aligned microfibers. Each fiber bundle tract consists of 10 stacked aligned fibers where each PCL fiber is 10 µm in diameter with the total height of the fiber walls 106 ± 15 µm (indicated as a white bar). The interfiber bundle distance is 200 µm.

Previously we demonstrated that 8-chamber radial structures using melt electrowriting and filled with different concentrations of 3D matrices were important tools to determine the optimal matrix and concentration for growth of the U87 human cell line [48]. In a different study, aligned solution electrospun nanofiber sheets enhanced migration of U87 cells, xenografted in mice, away from the primary brain tumor site to an extracortical site [49]. Thus, tailoring melt electrowritten printed fiber structural designs is helpful to address biological functions of tumor cells. Here we designed melt electrowritten 3D box-pore scaffolds or aligned microfibers to resemble axons and blood vessel tracts (Figure 1). Scanning electron microscopy measurements for both scaffolds and aligned microfibers consisted of 10 layers of single aligned PCL microfibers with an average fiber diameter of 10 µm, an inter-fiber bundle distance of 200 µm and an overall height of approximately 100 µm (Figure 1B,C). Additionally, for scaffolds, ten layers of microfibers were deposited in each direction (0° and 90°) resulting in a 20-fiber overlap at intersections (Figure 1B).

### 3.2. U87 Cells Show an Enhanced Scaffold Directed Durotaxis, Adhesion and Network Formation

**Figure 2:**
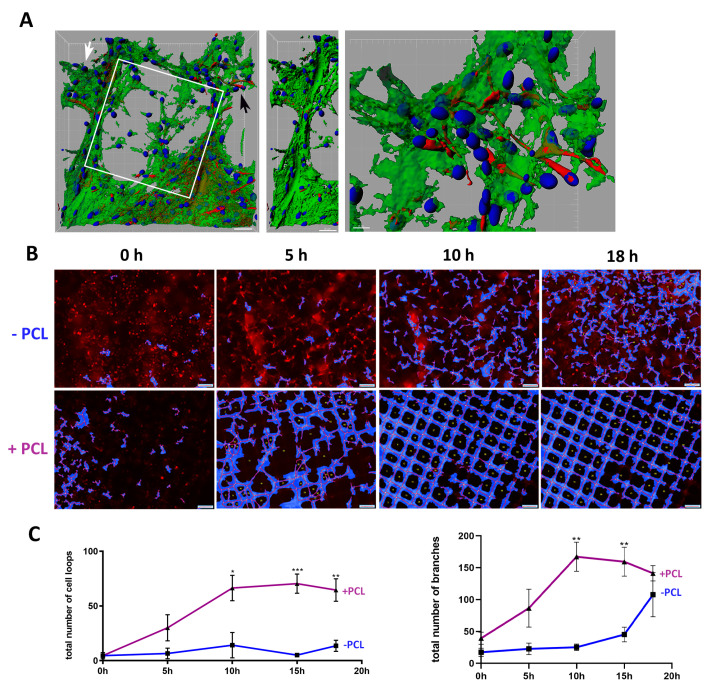
U87 durotaxis and cell network formation with scaffolds in 3D cultures. (**A**) Left image of a 3D reconstruction of an immunocytochemical staining of U87 cells at day 6 with 4.5 mg mL^−1^ Matrigel and a single box pore (200 µm) scaffold (white solid square) (white scale bar = 30 µm) (ß-tubulin, green; MAP2, red; Hoechst 33342, blue nuclei). The middle image shows the same region from the left image (white arrow) but without the scaffold to show the wrapping of U87 cells around the aligned microfibers. The right image is a magnification of the left image (black arrow) to demonstrate U87 cell interactions at day 6 (right scale bar = 10 µm) (ß-tubulin, green; MAP2, red; Hoechst 33342, blue nuclei). (**B**) Cell network formation assay. U87-td-farnesyl expressing cells grown in 3D with 4.5 mg mL^−1^ Matrigel alone (minus (–)PCL above) or with a scaffold (+PCL bottom) and imaged over time up to 18 h. Images were analyzed for cell branches or loop-like structures (blue with red lines) using the WimTube computer program. White scale bar = 200 µm. (**C**) Graphs show cell network formation assay (+PCL) scaffolds top red line (*n* = 6 experiments) and (-PCL) scaffolds bottom blue line (*n* = 8 experiments) quantified as the total number of cell loops (left graph, Y-axis) and branches (right graph, Y-axis) formed over 18 h. Significance was found comparing +PCL to -PCL; cell loops at 10 h * *p* = 0.0113; 15 h *** *p* = 0.0007; 18 h ** *p* = 0.0023; cell branches at 10 h ** *p* = 0.002; 15 h ** *p* = 0.0079).

Since GBM, and possibly breast tumor cells, can disseminate throughout the brain via axons and blood vessels, we first tested if U87 cells have an affinity for the scaffolds. Growing U87 cells in 3D with Matrigel (4.5 mg mL^−1^) and laminin-coated scaffolds resulted in an increased cellular affinity for the scaffold within 24 h and continued until 72 h (Figure S1). We observed at 24 to 72 h distinct amoeboid cells, migrating towards and associated with the scaffold (Figure S1A). By day six, U87 cells became more extended, where they formed distinct cellular networks between the scaffolds, but were also wrapped around aligned microfibers (Figure 2A and Figure S1A). In order to directly test an affinity of GBM cells for scaffolds, we live-imaged and tracked U87-td-farnesyl expressing GBM cells, which emit a red cytosolic fluorescence, in a time kinetic manner up to 18 h in Matrigel with laminin-coated scaffolds or Matrigel alone (Figure 2B, Videos S1 and S2). In the presence of scaffolds, tumor cells actively migrated toward and adhered to the scaffold forming loop and branch-like cellular networks (Figure 2B,C). In contrast, although U87 cells migrated throughout the Matrigel alone, no loop cellular networks were noted. Additionally, cell attraction appeared to occur equally along aligned microfibers and at the corners of scaffold structures, thus showing no topographical preference for a particular scaffold region. The total number of U87 cellular loops and branches in the presence of the scaffold significantly peaked at 10 h and 15 h compared to Matrigel alone (Figure 2C).

Previously it was shown that the stiffness of Matrigel (at 4.5 mg mL^•1^) represents a soft matrix of <100 Pa (Young’s modulus), but scaffolds exhibited a high stiffness of ~15 kPa [41,42]. A combination of hydrogel with scaffolds positively influences the overall handling by increasing stiffness synergistically [41,42]. In general, a higher matrix rigidity activates durotaxis-directed cell migration towards stiffer substrates [50,51]. Using the same conditions as above for Matrigel and scaffolds, we interpret our findings that a durotactic tumor cell response is occurring, where U87 cells migrate from a lower stiffness toward a substrate of a higher stiffness, where scaffolds having aligned microfibers represent a strong physical cue (Figure 1 and Figure 2). However, our findings demonstrating the wrapping of U87 cells around melt electrowritten fibers also suggest that the fiber topography affects cell behavior (Figure 2A).

### 3.3. GBM and Breast Cancer Cell Migratory Behavior with 3D-Aligned Microfibers

**Figure 3:**
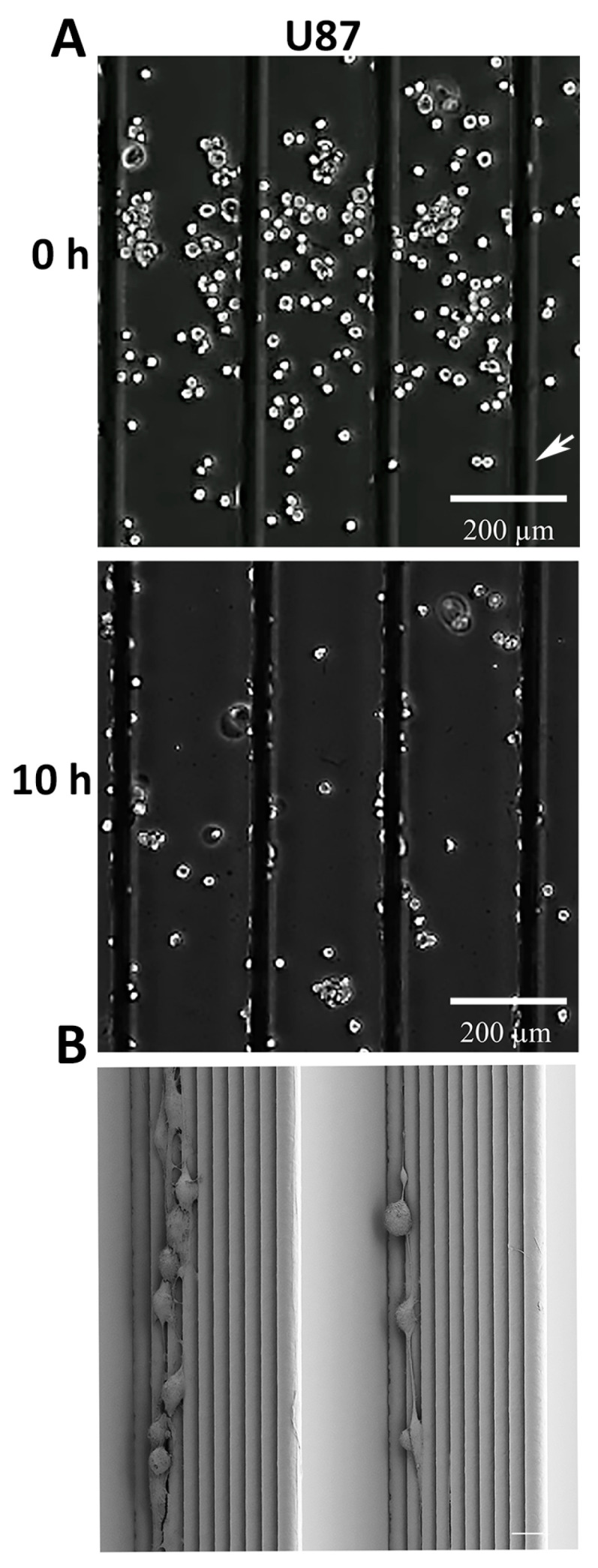
U87 durotaxis and cell migration with aligned microfibers. (**A**) Representative images of U87 cells on laminin-coated aligned microfibers at 0 h and 10 h; scale bar = 200 µm. (**B**) Representative scanning electron microscopy image of U87 cells on stacked aligned microfibers (total of 10 melt electrowritten printed fibers: scale bar = 20 μm).

**Figure 4:**
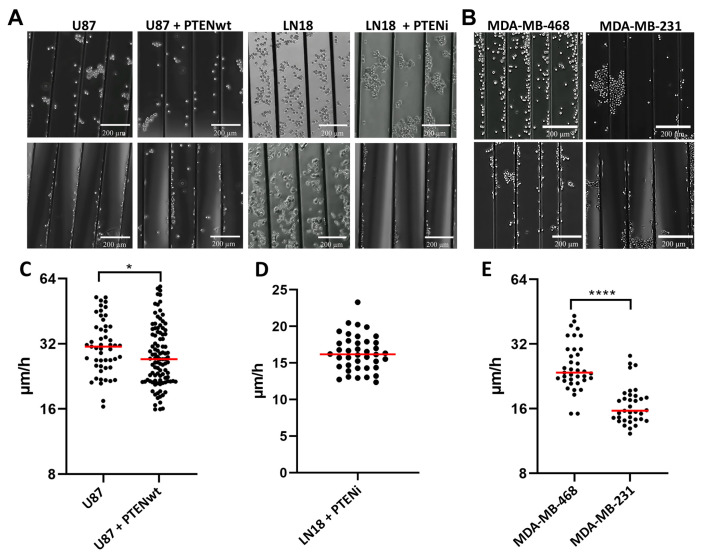
PTEN regulates GBM and TNBC cell durotaxis and migration. (**A**) Representative images from left to right of U87 PTEN loss of function cells (1st column, control) and U87 cells after *PTEN* wt transfection and rescue (2nd column) on laminin-coated aligned microfibers at 0 h (top) and 10 h (bottom) (scale bar = 200 µm). LN18 PTEN wt cells (3rd column, control), and LN18 cells (4th column) following PTEN inhibitor (PTENi) SF1670 treatment on laminin-coated aligned microfibers at 0 h (top) and 10 h (bottom). (**B**) Representative images of MDA-MB-468 PTEN loss of function cells and MDA-MB-231 PTEN wt cells on laminin-coated aligned microfibers at 0 h (top) and 10 h (bottom) (scale bar = 200 µm). Note the specific collective adherence at 0 h of MDA-MB-231 PTEN wt cells. (**C**) Aligned microfiber migration speed (μm/h) of U87 PTEN loss of function cells (control) (*n* = 4 experiments; *n* = 51 cells) and U87 cells after *PTEN* wt transfection and rescue (*n* = 3 experiments; *n* = 101 cells) * *p* = 0.0341. (**D**) Migration speed (μm/h) of LN18 PTEN wt cells with PTEN inhibitor (PTENi) SF1670; (*n* = 7 experiments; *n* = 40 cells). (**E**) Aligned microfiber migration speed (μm/h) of MDA-MB-468 PTEN loss of function cells (*n* = three experiments; *n* = 37 cells) and MDA-MB-231 PTEN wt cells (*n* = four experiments, *n* = 35 cells) **** *p* < 0.0001.

Following our findings demonstrating a strong durotactic response of U87 cells for scaffolds, we next implemented aligned microfibers (Figure 1C) to analyze the cell migratory behavior of GBM and TNBC cell lines in detail using live cell imaging (Figure 3 and Figure 4). We first tested U87 cell migration on aligned microfibers coated with laminin-111 (Figure 3 and Figure S1B), as laminin-111 is the most prominent brain ECM subtype and also enriched on small blood vessels [9,52]. Similar to scaffolds, an enhanced durotactic response of U87 cells was also observed for aligned microfibers as well as active cell migration (Figure 3A, Video S3). Furthermore, scanning electron microscopy indicated that most U87 cells were morphologically round and organized onto single microfibers supporting a topographical cue for movement (Figure 3B).

In striking contrast to U87 cells, LN18 cells showed no active durotactic response for laminin-coated aligned microfibers, and no migration (Figure 4A, Video S4). However, over a 10 h period some cells could adhere to aligned microfibers but did not migrate. We hypothesized that these differences between U87 and LN18 cells could be due to their opposite *PTEN* genotypes (U87 PTEN loss of function with no PTEN protein expression; LN18 PTEN wt) (Figure S2A,B). Based on the known regulatory role of PTEN in migration, we performed live cell imaging experiments modulating PTEN function. Transfecting and rescuing U87 cells (PTEN loss of function) with a *PTEN* wt overexpressing vector (Figure S2B) significantly blunted migration speed from 32.4 µm/h to 29.5 µm/h (*p* = 0.0341) (Figure 4C. In contrast, treatment of LN18 cells (PTEN wt) with a PTEN inhibitor restored a durotactic response, adherence and migration on laminin-coated aligned microfibers (Figure 4A, Video S5). A total rescue of cell migration with a speed of 16.4 µm/h, compared to no migration without inhibitor treatment, was found (Figure 4D).

We next tested if two TNBC cell lines with opposing *PTEN* genotypes behaved similar to GBM cells for durotaxis, cell adhesion and migration, using the same experimental conditions. Results showed that MDA-MB-468 cells (PTEN loss of function) and MDA-MB-231 cells (PTEN wt) were comparable to GBM cell lines, but with some differences (Figure 4B,E). For example, MDA-MB-468 cells (PTEN loss of function) like U87 cells demonstrated a strong durotactic response for aligned microfibers, cell adhesion and migration (Video S6). Although both PTEN wt, in contrast to LN18 and MDA-MB-231 cells, showed more collective cell adhesion, migration only occurred as single cells (Video S7). However, MDA-MB-231 migrated significantly slower at 17 µm/h on aligned microfibers than MDA-MB-468 (PTEN loss of function) at 26.5 µm/h (*p* < 0.0001) (Figure 4B,E).

### 3.4. RHOB as a Novel Regulator of Migration

**Figure 5:**
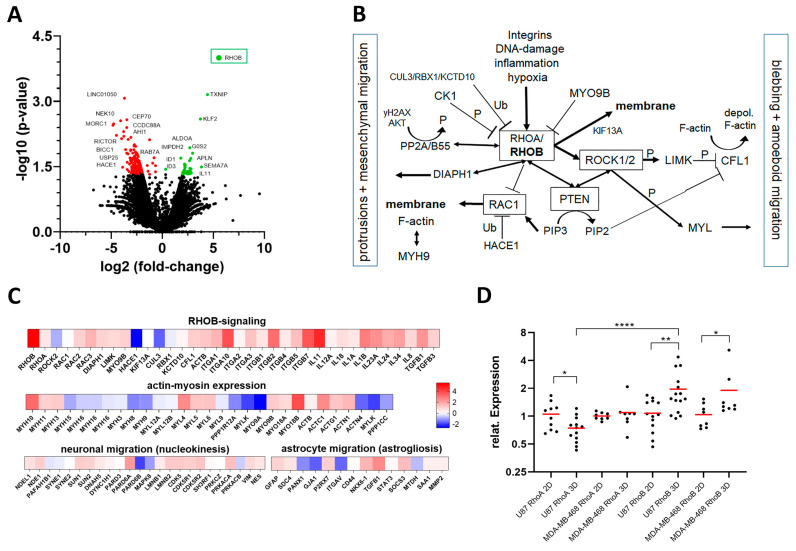
RHOB signaling and regulation of migration. (**A**) RNA-seq data (Volcano-plot) of U87 PTEN loss of function cells grown in 3D with Matrigel and scaffolds versus 2D (3 days). Red dots (left): significantly (*p* < 0.05) down regulated genes; green (right) dots significantly (*p* < 0.05) up-regulated genes. *RHOB* was 5.528-fold log2 up regulated in U87 3D cultures and scaffolds (*p* = 0.0001). Other significantly up-regulated genes include *G0S2* (6.4-fold, *p* = 0.011), induced in glioma with high invasion [55], *APLN* (4.55-fold, *p* = 0.0307) induces colon cancer cell migration [56], *ALDOA* (3.37-fold, *p* = 0.0202) promotes lamellipodia [57], *SEMA7A* (3.84-fold, *p* = 0.05) via ITGB1 [58] and *IMPDH2* (1.25-fold, *p* = 0.036) regulates colorectal cancer cell migration [59] (Supplemental Tables S1 and S2). (**B**) Overview of the RHOB signaling pathway. RAC1 promotes an elongated cell phenotype (mesenchymal) migration (left), which is inhibited by RHOB. RHO activates ROCK leading to blebbing and amoeboid migration (right) and is negatively regulated by PTEN dephosphorylating phosphatidylinositol-3,4,5-triphosphate (PIP3) to phosphatidylinositol-4,5-bisphosphate (PIP2). Ub (ubiquitinylated); P (phosphorylated). (**C**) RNA-seq gene expression differences (heatmaps) of U87 PTEN loss of function cells in 3D with Matrigel and scaffolds versus U87 in 2D (red = log2-fold up regulated and blue= log2-fold down regulated). Top heat map: RHOB signaling genes, e.g., up-regulation of Integrins (esp. *ITGA10* and *ITGB2*) and inflammation genes (e.g., *IL11*, *IL1B*) stimulating RHOB; middle heatmap: actin-myosin expression; bottom heatmap: neuronal migration (left) and astrocyte migration (right). (**D**) QPCR and relative gene expression (2-DDCt) of *RHOA* and *RHOB* in U87 and MDA-MB-468 PTEN loss of function cells in 2D vs. U87 and MDA-MB-468 cells in 3D with Matrigel and scaffolds (3 days). Significant values from left to right: U87 *RHOA* expression in 2D vs. 3D: * *p* = 0.0316; U87 *RHOB* expression in 2D vs. 3D: ** *p* = 0.0031, MDA-MB-468 *RHOB* expression in 2D vs. 3D: * *p* = 0.0409. U87 *RHOA* 3D vs. U87 *RHOB* 3D: **** *p* < 0.0001.

To further explain PTEN regulation of GBM and TNBC cell migration, we focused on the RHO-ROCK signaling pathway (Figure 5B). Next to RHOB and RHOC, RHOA has been described as the most prominent regulator of the migration signaling pathway [53]. To gain insight into the genes including RHO-ROCK signaling and other pathways, we performed RNA-seq from U87 cells grown in 3D with Matrigel and scaffolds compared to 2D controls. Results showed a highly significant 46.15-fold increase of RHOB expression in 3D versus 2D (*p* = 0.0001) (Figure 5A, Table S1). Differential gene expression of RHOB signaling members, as well as actin and myosin genes, support regulation of migration (Figure 5C). On the other hand, genes involved in other migration modes, such as neuronal migration via nucleokinesis (nuclear piston migration) and astrocyte migration (astrogliosis) were not increased, supporting the hypothesis that GBM cell migration is predominantly regulated by RHO/ROCK signaling (Figure 5C) [54].

The significant role of RHOB was validated using quantitative real time PCR (qPCR) for both U87 and MDA-MB-468 cells using the same 3D and 2D culture conditions as for RNA-seq (Figure 5D). Furthermore, RHOB expression was significantly higher than RHOA for U87 cells in 3D. In contrast, both PTEN wt LN18 and MDA-MB-231 cells showed no significant increase of RHOB gene expression compared to 2D (Figure S2C). Importantly, U87 cells in 3D Matrigel without PCL also showed a significant induction of RHOB, but not RHOA expression, compared to U87 cells seeded on 2D or 2D Matrigel coating (Figure S2D). In contrast, LN18 showed no induction of RHOB expression in 3D Matrigel alone. Our results support that tumor cells with a PTEN loss of function induce high levels of RHOB expression and enhanced migration dependent on a 3D environment; however, durotaxis is only active in the presence of PCL (Figure 2B,C).

**Figure 6:**
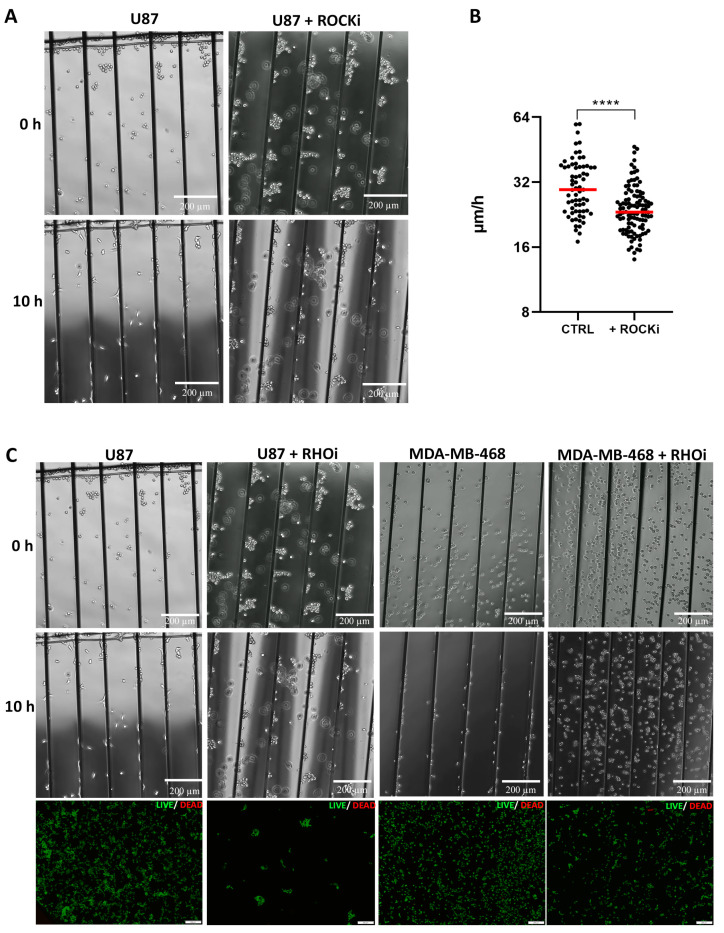
RHO-ROCK regulates tumor cell durotaxis and migration. (**A**) Representative images of U87 PTEN loss of function control cells (left) or cells treated with the ROCK inhibitor (ROCKi) Y-27632 at 0 h and 10 h on laminin-coated aligned microfibers. (**B**) Migration speed (μm/h) of U87 cells (control, CTRL) (*n* = 7 experiments; *n* = 67 cells) vs. U87 cells with the ROCK inhibitor (ROCKi)Y-27632 (*n* = 9 experiments, *n* = 107 cells); **** *p* < 0.0002. (**C**) Representative images (from left to right) of U87 control (1st column) or cells treated with RHO inhibitor (RHOi) (2nd column) and MDA-MB-468 PTEN wt cells control (3rd column) or treated with the RHO inhibitor (RHOi) (4th column) on aligned microfibers coated with laminin at 0 h and 10 h (U87: *n* = 4 experiments; MDA-MB-468: *n* = 3 experiments). Lower panels are examples of cell viability analyses of the respective tumor cells above after RHO inhibitor treatment. Green: live cells; red: dead cells.

Based upon our RNA-Seq and qPCR results we initiated functional cell culture studies to further unravel the role of RHOB and pathway members in the migratory behavior of U87 and MDA-MB-468 cells (Figure 5B). Treatment of U87 cells with a ROCK inhibitor demonstrated a highly significant decrease of migration speed to 24.5 µm/h (*p* < 0.0001) on aligned microfibers compared to control, where cells migrated at an average speed of 31.5 µm/h (Figure 6A,B). Further in line with RHO/ROCK signaling, treating both PTEN loss of function U87 and MDA-MB-468 cells with a RHO inhibitor blunted durotaxis and adhesion, and completely inhibited cell migration, while both cell types remained viable (Figure 6C, Video S8). This result supports that blocking RHO kinase primarily inhibits cell migration, which is also necessary for the durotactic response.

**Figure 7:**
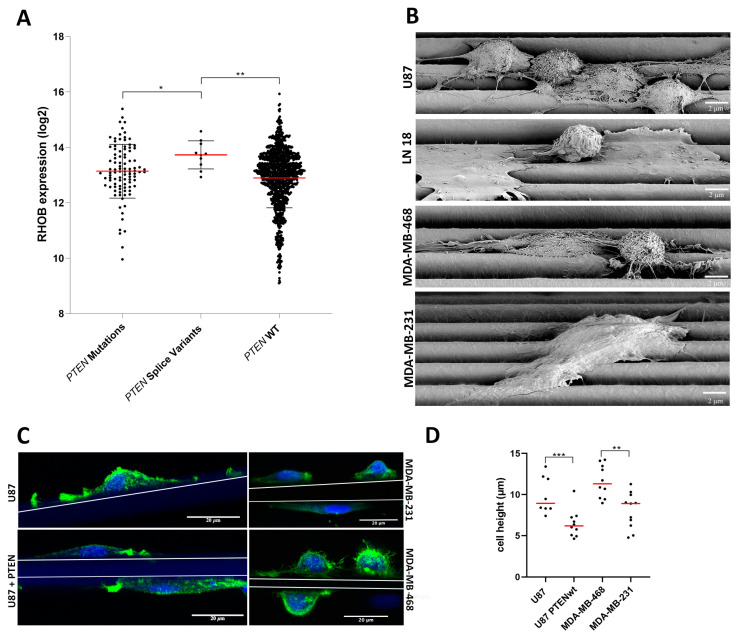
RHOB signaling in primary GBM and breast tumors and RHOB regulation of cell morphology. (**A**) Correlation of *PTEN* wt or *PTEN* splice or other mutations with *RHOB* expression from primary GBM and breast cancers (TCGA); * *p* = 0.031; ** *p* = 0.003; *PTEN* mutations: *n* = 98 tumors; including GBM (*n* = 46 tumors) and breast cancer (*n* = 52 tumors). *PTEN* splice variants: *n* = 10 tumors; including GBM (*n* = 4) and breast cancer (*n* = 6). *PTEN* wt: *n* = 1102 tumors including GBM (*n* = 97) and breast cancer (*n* = 1005 tumors). (**B**) Representative scanning electron microscopy images on laminin-coated aligned microfibers with U87 PTEN loss of function, LN18 PTEN wt, MDA-MB-468 PTEN loss of function and MDA-MB-231 PTEN wt cells at 24 h after seeding (scale bar = 2 µm). (**C**) Confocal z-stack images of F-actin Alexa 488 staining of U87 PTEN loss of function control cells (top left) vs. U87 cells after *PTEN* wt transfection and rescue (bottom left); and MDA-MB-231 PTEN wt (top) vs. MDA-MB-468 PTEN loss of function cells (bottom) on laminin-coated aligned microfibers at 24 h after seeding (green: F-actin; blue: DRAQ5 indicates nuclei) (scale bar = 20 µm). (**D**) Quantification of cell height from confocal images (µm) (Y-axis) of U87 PTEN loss of function (control) vs. U87 after *PTEN* wt transfection and rescue and MDA-MB-468 PTEN loss of function vs. MDA-MB-231 PTEN wt cells. *** *p* = 0.0010; ** *p* = 0.0016.

Finally, we asked if there was a clinical correlation between primary GBM and breast cancers with *PTEN* wt or *PTEN* splice or other mutations and RHOB expression using The Cancer Genome Atlas (TCGA). Although rare, primary GBM and breast tumors with *PTEN* splice mutations had significantly higher RHOB gene expression versus tumors with *PTEN* wt or other *PTEN* mutations (Figure 7A). Upon further analyses, in seven of ten of the primary GBM and breast cancer tumors with *PTEN* splice mutations and high RHOB expression a homozygote *PTEN* loss of function was found. In line with TCGA primary tumors, both U87 and MDA-MB-468 cells harbor *PTEN* splice variants leading to a homozygote PTEN loss of function (Figure S2A for U87). In contrast, analyzing RHOA expression in primary GBM and breast tumors, using the same TCGA cohorts, showed no significant RHOA expression for tumors with *PTEN* splice variants. However, a significant higher expression of RHOA (~10%) was found in tumors with *PTEN* mutations versus tumors with *PTEN* wt (*p* = 0.001). The above findings, along with our cell culture studies, support a prominent role for RHOB and *PTEN* splice variants with PTEN loss of function in regulating tumor cell migration of GBM and breast cancers.

### 3.5. RHOB Signaling as a Regulator of Cell Morphology

A more in-depth view of RHOB signaling as regulated by PTEN supports a cellular decision of either an amoeboid or mesenchymal cell shape as well as movement (Figure 5B). Microscopic analyses comparing both GBM and TNBC cell lines with opposing *PTEN* genotypes on laminin-coated aligned microfibers noted significant differences in cell morphology. For example, scanning electron microscopy demonstrated that high RHOB-expressing U87 and MDA-MB-468 cells with *PTEN* loss of function were amoeboid and primarily located mainly on single fibers (Figure 7B). In contrast, both low RHOB-expressing and PTEN wt LN18 and MDA-MB-231 cells were flat and spread over 2–3 fibers. Measuring the cell heights confirmed that amoeboid U87 and MDA-MB-468 cells had average cell heights of 9.9 µm and 12.2 µm, respectively (Figure 7D). These values were significantly higher compared to MDA-MB-231 *PTEN* wt cells and U87 cells transfected with a *PTEN* wt overexpressing vector, which had average cell heights of 8.3 µm and 6.5 µm, respectively.

**Figure 8:**
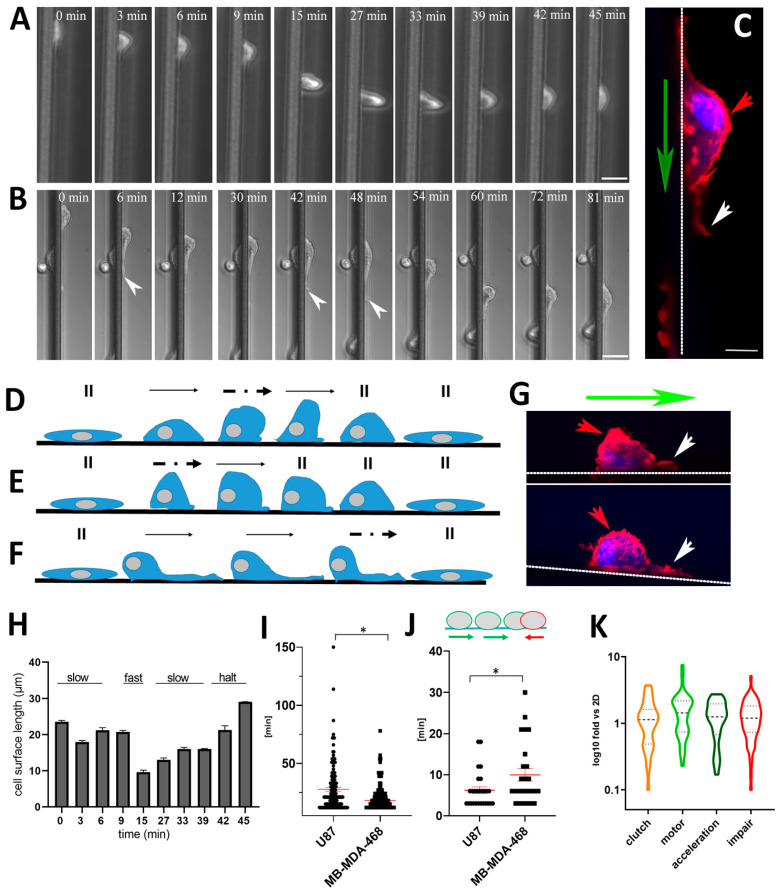
Skating snail-like cellular plasticity modes and persistence on aligned microfibers. (**A**,**B**) Consecutive images of U87 cell plasticity modes and migration over time; white arrows indicate leading edge cellular protrusions (scale bar = 25 µm). (**C**) Confocal image of z-stacks showing F-actin Red staining of an amoeboid U87 PTEN loss of function cell (red: F-actin, blue DRAQ5 indicates nuclei), red arrow indicates cell nucleus at cell rear, white arrow indicates leading edge cellular protrusion, green arrow indicates direction of movement (scale bar = 10 µm). (**D**–**F**) Schematic images of migration cell plasticity modes of “skating snail-like” movements for U87 cells. II = nonmoving resting cells; thin arrow = slow cell movement; bold dotted-thick arrow = fast cell movement (**G**) Confocal image of z-stacks of two U87 cells (top, bottom) (red: F-actin, blue DRAQ5 indicates nuclei), red arrow indicates strong F-actin stain around the cell circumference, white arrow indicates cellular protrusion, and green arrow indicates direction of movement. (**H**) Quantification of cell surface length on aligned microfibers (Y-axis) of different U87 migration modes (X-axis). Shortest bar graph = fastest mode of movement. (**I**) Quantification of total U87 and MDA-MB-468 cell migration time (min) for single cell movements along aligned microfibers, FigU87: 27.06 ± 1.65 min; MDA-MB-468: 21.08 ± 1.09 min; * *p* = 0.0142. (**J**) Quantification of reverse migration (min) of single movements of U87 and MDA-MB-468 after continual forward movements (green circle and arrow = forward movement and red circle and arrow = reverse movement), U87: 6.22 ± 0.83 min, MDA-MB-468: 9.96 ± 1.53 min; * *p* = 0.0462. A total of 258 single cell movements were analyzed for both 8I and 8J. (**K**) RNA-seq gene expression differences of U87 cells in 3D with Matrigel and scaffolds versus U87 in 2D. Expression of clutch genes (*n* = 35), motor genes (*n* = 40), acceleration genes (*n* = 50) and migration impairment genes (*n* = 58) [60,61,62].

Using confocal laser microscopy, detecting F-actin confirmed cell morphologies as observed by scanning electron microscopy (Figure 7B,C). Congruent with an amoeboid morphology, U87 cells and MDA-MB-468 cells showed distinct F-actin protrusions with different sizes at the leading edge, but also small actin blebs on the surface of MDA-MB-468 cells (Figure 7C and Figure 8C,G with white arrows). Prominent F-actin (Phalloidin positive) staining was also noted at the cell surface for both cell lines along the membrane with different intensities (Figure 7C and Figure 8C,G with red arrows). The nucleus of the U87 cells was primarily located at the rear of the cell, opposite to the direction of movement (Figure 7C and Figure 8C).

### 3.6. Skating Snail-Like Cell Migration and the Motor Clutch Model

To unravel a more detailed analysis of the migration mode, we focused on U87 and MDA-MB-468 tumor cells with PTEN loss of function and high RHOB expression. Using live cell imaging with a higher magnification, we compared cell morphology, plasticity modes, the total migratory distance and persistence behavior on aligned microfibers. Distinct amoeboid plasticity modes resembling “snail-like” crawling, followed by alternating fast movements, was common for both tumor cell types (Figure 7C, Figure 8A–G and Figure S3, Videos S9 and S10). These skating snail-like cellular forms resemble how speed skaters on ice initially propel their forces forward to accelerate and then skate in a gliding manner with higher speed.

For example, one mode showed that skating snail-like U87 cells were initially slow, then accelerated (between 9–15 min) and slowed down again (between 30–42 min) (Figure 8A,D,G top, Figure S3B). Another skating snail-like U87 cell mode demonstrated an instant acceleration to a fast mode then slowed down and stopped (Figure 8E,G bottom; Figure S3B). Lastly, a skating snail-like U87 cell mode showed a slow “crawling” and then a faster sliding with long cellular protrusions at the leading edge (Figure 8B,C,F and Figure S3B). These changes in cellular plasticity modes dramatically point to an intrinsic characteristic of these tumor cells. However, when cells paused their migration on aligned microfibers, both U87 and MDA-MB-468 cells displayed a more elongated and flattened morphology (Figure 8A,B,D–F and Figure S3A,B). Analyzing the cell surface length of moving cells on aligned microfibers demonstrated that cells during fast movements had the shortest length compared to slow and halted cells (Figure 8H). Next, we addressed the total migratory distance and persistent migration of U87 and MDA-MB-468 cells. Comparing the total time that cells migrated on aligned microfibers revealed that U87 cells significantly migrated for longer times (27.06 ± 1.65 min) compared to MDA-MB-468 cells (21.08 ± 1.09 min) (Figure 8I). Addressing the total time of reverse migration occurring in one single cellular movement, U87 cells demonstrated significantly fewer reverse movements (6.22 ± 0.83 min) compared to MDA-MB-468 cells (9.96 ± 1.53 min) (Figure 8J). The above findings support that U87 cells significantly migrated more persistently than MDA-MB-468.

The motor clutch model describes the physical and molecular mechanics of how cells move on substrates [63]. This complex process involves actin polymerization at the leading cell edge driving forward cell movement, which is coordinated with actin depolymerization proximally and centrally along with localized myosin motors that pull and exert force on this F-actin network, resulting in retrograde actin flow [64]. Clutch adaptor proteins such as cell receptor integrins and other clutch components, such as talin and vinculin, interact with F-actin, transmit forces to adhesion sites and regulate the cellular grip on the extracellular matrix controlling cell movement [64,65]. Previously it was shown that the molecular processes describing the motor-clutch model implemented many different genes [60,61,62] (*n* = genes) for clutch (*n* = 35), motor (*n* = 40), acceleration (*n* = 50) and migration impairment (*n* = 58). In line with the motor clutch model, we analyzed the relevant proteins mediating the motor clutch mechanism, as well as proposed proteins responsible for acceleration and impairment of migration according to our RNA-seq data [60,61,62]. Although some motor clutch specific genes were found more highly expressed in U87 grown in 3D with Matrigel and laminin-coated scaffolds and 2D cultures (Figure 5C), our expression analyses using RNA-seq of the above 183 genes showed no significant differences (Figure 8K).

## 4. Discussion

One of the first comparative histological analyses by Scherer in 1938 showed that GBM growth and dissemination throughout the brain depends on architectural organization [15]. Histologically, Scherer discovered that GBM cells were mainly associated with white matter axon bundles, and could even follow dendrites when invading the cortex. Importantly, secondary tumors to the brain were mainly detected in border zone regions, such as the cerebral vascular supply and gray and white matter junctions, further supporting that metastatic tumor cells travel along the arterial tree [66]. Brain structures in terms of stiffness are still under investigation. For example, local viscoelastic properties of neurons and glial cells using scanning force microscopy showed that the elastic storage modulus (E’) of astrocytes (glial cells) was between ~300 Pa (30 Hz) to ~520 Pa (200 Hz), whereas that of neurons ranged from ~650 Pa (30 Hz) to ~1,590 Pa (200 Hz) [67]. These values defined the cells of the central nervous system (CNS) as very soft compared to other cells, such as fibroblasts (E′ ~3 kPa at 200 Hz) [68]. Axon bundle tracts have widths ranging from 0.1–10 μm [69] and stiffnesses recorded of 4.6 ± 1.5 kPa (Young’s modulus) [70]. Interestingly, using waveguide elastography with healthy individuals’ *in vivo*, similar values for white matter tracts of 4–4.6 kPa (shear modulus) were found [71].

Evaluation of stiffness of arteries and veins is ongoing in the literature; however, stiffness analyses of brain vessels are still needed. For example, the Young’s elastic modulus of human arteries has been measured as 679 kPa ± 304 kPa standard deviation with an adjusted arterial diameter of the left carotid artery of 410 µm ± 130 µm standard deviation (*n* = 6441 individuals) [72]. Diameters of smaller human blood vessels are ~8 μm for capillaries and 20–30 μm for venules and arterioles using magnetic resonance imaging with computed tomography [73]. Multiphoton laser-scanning microscopy of mice brains showed that the brain vessels ranged between ~1 and 20 µm in diameter [74]. Most importantly, using live imaging of GBM cells invading and growing in mice brains, it was shown that GBM cells migrated primarily along blood microvessels of the peritumoral area, especially when microvessels ran parallel to each other [74].

Isolated human normal brain and GBM have similar shear storage moduli at a low strain (elastic moduli of ~300 Pa) [75]. However, normal brain and GBM shear moduli increased to kPa range when uniaxially compressed ex vivo [75]. ECM protein components are increased in GBM, resulting in higher stiffness [76]. In contrast to the above, magnetic resonance elastography (MRE), which measures stiffnesses via mechanical shear waves in vivo, showed that GBM is softer than normal brain [77]. However, MRE results especially of GBM, have to be critically evaluated due to different cerebral blood flows, and lower stiffness measurements could reflect the overall heterogeneity including hemorrhaging, edema and necrosis [78]. Importantly, vascularization and interstitial pressure are very high in GBM (and other tumors), due to the fact that GBM shows the highest microvascular proliferation and microvessel density of all brain cancers [79]. What is clearly needed are measurements of localized solid tumor regions away from necrosis and edema to assess solid tumor stiffness. Thus, it is highly likely that GBM has essentially a higher stiffness then normal brain *in vivo*.

It is well known that breast cancer tumors are stiffer (E’ = 4.04 ± 0.9 kPa) than normal mammary tissue (E’ = 0.167 ± 0.031 kPa) [80] supporting tumor cell mediated ECM remodeling. On the other hand, single primary breast cancer cells are very soft (~300 Pa), similar to the CNS cells described above [81]. It is conceivable that tumor cells with different mechanical conditioning target different metastatic sites, as shown for breast cancer cells [82]. Since it is known that cells have a high affinity for stiffer substrates [17], it is plausible that tumor cells demonstrate a durotactic activity for stiffer white matter tracts and blood vessels in the brain. Support for this stems from our experiments, where both TNBC and GBM cells with PTEN loss of function demonstrated enhanced affinity for aligned microfiber tracts seeded in a nonstiff fluid environment. This scenario could especially represent circulating TNBC cells entering the blood/brain barrier and thus seeking stiffer substrates.

In the present study, we implemented scaffolds and aligned microfibers (each 10 μm) with measured compression stiffnesses of ~15 kPa including porous spaces [41] to simulate axon tracts and blood vessels within the brain. Although, the aligned microfibers used in this study are in line with the diameters of axon bundles and small blood vessels and ECM components, the high stiffness of microfibers may be relevant with small blood vessels but higher than axons measured in normal brain. However, axon stiffness in the GBM tumor environment with increased levels of ECM is unknown. Importantly, we present novel findings demonstrating that both GBM and TNBC cells with homozygote *PTEN* loss of function and high RHOB gene expression show similar 3D migration properties in contrast to GBM and TNBC cells with PTEN wt and low RHOB expression. Furthermore, using TCGA analyses, 70% of primary GBM and breast cancer tumors with *PTEN* splice mutations had homozygote *PTEN* loss of function with significantly high RHOB expression (Figure 7A). These primary tumors are similar to the cell lines used in this study, supporting the clinical relevance of our experimental findings regarding 3D migration. Thus, we conclude that the status of a specific tumor cell genotype together with expression of RHO/ROCK-signaling genes represent “molecular cues” for 3D structures regulating durotaxis, cell adhesion and migration.

Only GBM and TNBC cells with *PTEN* loss of function and high RHOB gene expression resulted in an enhanced single cell durotactic response, promoting cells to migrate towards stiffer scaffolds or aligned microfibers. Furthermore, following adherence these tumor cells significantly had a faster migration speed on aligned microfibers than PTEN wt and low RHOB expressing cells. Recently, two other GBM cell lines (U251 and GL15), with either a PTEN mutation or a deletion, also demonstrated durotaxis with a polydimethylsiloxane substrate [83]. It is noteworthy that MDA-MB-231 cells with PTEN wt and low RHOB expression showed a specific mode of “collective cell adherence” with aligned microfibers, where single cells could load onto and migrate at a slower speed. In contrast no durotaxis or migration occurred with LN18 PTEN wt. Interestingly, an essential role for *Pten* controlling collective cell migration was also found during mouse embryonic development [84]. It was demonstrated that TGFB induced migration via PTEN suppression, where TGFB1 switched breast cancer cells from a collective to a single cell migration mode via RHO/ROCK signaling [85]. It will be important to further study the collective nature of MDA-MB-231 binding to aligned microfibers.

Another study using solution electrospun nanofiber scaffolds with a higher stiffness, of up to 166 kPa, demonstrated increased migration of glioma stem cells depending on multibranched N-glycans metabolized by MGAT5 [86]. According to our RNA-seq data of U87 cells grown in 3D and scaffolds, with a ~10-fold lower stiffness to the above study, the MGAT5 gene as well as other MGAT genes were not overexpressed, implying that multibranched N-glycans were probably not responsible for migration. In contrast to glioma stem cells, U87 cells showed no overexpression of stem cell markers, such as SOX2, ZEB1, ATXN1, ALCAM, CD9, ITGA7, CD44 and CHI3L1.

Although much is known regarding PTEN regulating chemotaxis and migration [87], we prove a pivotal role for PTEN controlling durotaxis and migration speed using aligned microfibers. Manipulating PTEN with a PTEN inhibitor, or rescuing PTEN function, completely recovered durotaxis and migration of LN18 PTEN wt cells or blunted cell speed of U87, respectively. The 2D studies of Tamura et al. showed that NIH3T3 mouse embryonic fibroblasts and U87 cells overexpressing *PTEN* lowered 2D migration via reduced integrin-mediated cell spreading and focal adhesions [88]. The latter study also showed that *PTEN* inhibition enhanced cell migration using 2D scratch assays. Furthermore, our findings reveal an essential role of RHO or downstream ROCK kinase activity for durotaxis and migration. Corroborating our U87 RNA-seq, where RHOB was the highest significantly expressed gene (46.15-fold), which was confirmed with qPCR for U87 and MDA-MB-468, inhibition of RHO halted both durotaxis and migration without cell death. In addition, inhibiting ROCK also significantly blunted migration speed. Another ROCK inhibitor, Fasudil^®^, reduced invasion of GBM cells xenografted into mice [89]. In contrast, using MDA-MB-231 cells in confined environments, such as 3 µm channels or GBM cells on 2D surfaces, showed no inhibition of migration and speed with ROCK inhibitor Y-27632, supporting no Myosin II involvement using these conditions [90,91]. Taken together, our above findings, along with the literature, support an essential regulatory role for the RHOB/ROCK/PTEN signaling pathway controlling durotaxis and migration.

Concerning cell morphology, Gong et al. showed that RHOB overexpression, along with RHOB shuttling from endosomes to the plasma membrane, led to cell blebbing and increased amoeboid migration in 3D collagen [35]. In contrast, a morphological switch from an amoeboid to a more elongated lamellipodial shaped cell involved RHOA signaling [54]. We also detected a significant switch of PTEN rescued U87 from an amoeboid shape to an elongated flat phenotype with a lower migration speed. Furthermore, we discovered that U87 and MDA-MB-468 cells with homozygote *PTEN* loss of function and increased *RHOB* expression had distinct amoeboid cell shapes migrating through a 3D matrix towards scaffolds (Figure S1A), and also on aligned microfibers (Figure 7B,C and Figure 8A–G). Further support for an amoeboid shape showed significant increased cell heights for U87 and MDA-MB-468 cells, compared to more elongated cells with PTEN wt and RHOB low expression. Normal mammary epithelial cells spread more on a stiff matrix (5000 Pa) compared to a soft matrix of 140 Pa, which was shown to be dependent on RHO, ROCK or Myosin [92].

Our confocal imaging of amoeboid shaped cells also showed enhancement of F-actin along the cell rim or cortex, which extended into different sized protrusions at the leading edge of the cell. The nucleus was mainly positioned in the cell rear. An enriched F-actin cortex has previously been noted in spherical-like shaped cells, presenting a single barrier along the cell circumference [93]. It is notable that RHOB recruits DIAPH1 to endosomes forming an F-actin coat [94]. It would be interesting to analyze if RHOB and DIAPH1 also play a role in F-actin membrane coating, due to the fact that RHOB shuttles between endosomes and plasma membrane [35]. Increased actin-myosin contraction also associates with highly motile round cancer cells along with decreased adhesion, thus supporting a basis for our observations of fast amoeboid cell movements on aligned microfibers with less cell surface area [95].

It is known that different cellular protrusions can be found associated with amoeboid-shaped cells, contributing to cell movement [96]. It was shown that single cellular focal adhesions autonomously sense the environment for stiffness and rigidity [17]. The latter study proved that the sensing of focal adhesions via the Paxillin/Vinculin/Focal adhesion kinase (PTK2) pathway was essential for durotaxis, but not chemotaxis. Our RNA-seq of U87 cells in 3D with scaffolds versus 2D showed an induction of the adhesion adaptor gene Paxillin (2.46-fold), but not of Vinculin and Focal adhesion kinase. Additionally, it was shown that soft matrices suppressed actin-myosin assembly and orientation. Specifically, myosin heavy chain IIA (MYH9) initiated formation of actin-myosin filaments and myosin heavy chain IIB (MYH10) bound and stabilized these filaments. In contrast to soft matrices, MYH9 did not polarize, but MYH10 was induced during durotaxis with stiff substrates [97], which we found to be 7.5-fold upregulated with our RNA-seq of U87 in 3D with scaffolds. Lastly, regarding the nucleus position of migrating cells, it was shown that the direction of cell migration appeared to be driven by positioning the nucleus towards the cell rear in multiple cell types via microtubules, with a possible extension of the leading edge [98]. Rearward orientation of the nucleus was also regulated by activation of RHO signaling pathways [99].

Our scanning electron microscopy findings showing alignment of single amoeboid cells on individual 10 µm fibers within a single 100 µm aligned microfiber tract, support a topographical cue for migration (Figure 3B and Figure 7B). On the other hand, elongated cells appeared to topographically sense and stretch over several PCL fibers. Although the majority of topotaxis studies address cells sensing specific 3D nanostructures [20], our laminin-coated aligned microfibers represent smooth 10 µm round tube-like structures. Lastly, for both amoeboid U87 and MDA-MB-468 cells with high RHOB expression and PTEN loss of function, we recorded different plasticity modes, which we coined “skating snails”. For example, U87 cell surface lengths along the aligned microfibers corresponded with different speeds. Fast migrating U87 cells showed a surface length of 10 μm, whereas slow and halted U87 cells were 20 µm or 30 µm in length, respectively (Figure 8H). In line with our findings, Maiuri et al. showed that cell speed and persistence are exponentially coupled via actin flow [100]. Although U87 and MDA-MB-468 showed similar plasticity modes, U87 cells were more persistent, “skating” for longer distances than MDA-MB-468 cells. Thus, we support the idea that these cellular plasticity modes reflect intrinsic genetic differences between GBM and TNBC cells.

Other publications have also implemented biomaterials or ECM fiber structures to mimic brain structures, such as axons, to investigate tumor cell migration in the brain [49,101,102,103]. All these publications tested fiber sizes, fiber widths, single, stacked or parallel fibers to resemble axon and blood vessel structures. One common finding among all studies was the striking different plasticity modes of cells migrating on various fiber constructs. Doyle et al. demonstrated that cells migrating on 1.5 µm single fibrillar lines (also called 1D) composed of fibronectin were identical to cells migrating in a 3D fibrillar matrix but were significantly slower on 2D fibronectin coated substrates [101]. The authors concluded that both 1D and 3D cellular migration were comparable based upon different properties of migrating cells. For example, cells had a uniaxial cell morphology with a single lamellipodia and a coordinated protrusion-retraction cycle, which resulted in a unidirectional rapid migration independent of ECM density, but dependent on myosin II contractility.

## 5. Conclusions

Our findings validate a significant role of specific *PTEN* genotypes along with RHOB signaling controlling cell morphology, durotaxis, adhesion, cellular plasticity and migration speed of tumor cells. For both GBM and TNBC types with homozygote *PTEN* loss of function and high RHOB expression, we propose that the cellular plasticity modes of amoeboid cells promote enhanced durotaxis, topotaxis, increased migratory speeds and longer traveling distances, which could lead to faster dissemination throughout the brain. The fact that PTEN regulates the cell polarity of the phosphatidylinositol-3,4,5-triphosphate/phosphatidylinositol-4,5-bisphosphate gradient supports the idea that an aberrant gradient due to PTEN loss of function could be responsible for the above deregulations. Presently clinical treatments have not yet achieved the goal of extending the survival of patients with GBM or breast cancer patients with brain metastasizing tumors. RHO GTPase inhibitors have been implemented in preclinical xenografted animal studies demonstrating anti-tumor effects [104]. Data from TCGA studies support that RHOA is mainly deleted among all major cancer types, thus being a possible tumor suppressor such as PTEN and would not be a proper candidate for inhibitors [105]. On the other hand, TCGA data supports RHOB as a proto-oncogene amplified in the majority of all major tumors, including GBM and invasive breast cancers [105]. Thus, RHOB could represent a target for therapeutic treatment. This opens the door for new therapeutic drugs regarding tumors overexpressing RHOB targeting cell migration. Our study demonstrates the importance of PTEN and RHOB not only for primary and metastasizing tumors, but also for circulating tumor cells upon entry into a stiffer environment, where RHOB inhibitors could play an essential role for improved therapy.

## Data Availability

The datasets used and/or analyzed during the current study are available from the corresponding author on reasonable request.

## Supplementary Materials

The following are available online at https://www.mdpi.com/article/10.3390/cancers13205144/s1. Figure S1: Durotaxis, topotaxis and cell adhesion/network formation on 3D scaffolds, Figure S2: PTEN splice mutation analysis and RHOA/RHOB expression in GBM and TNBC cells, Figure S3: The skating snail-like cellular plasticity modes of migration, Figure S4: Uncropped Figure S2B (upper), Figure S5: Uncropped Figure S2B (down), Table S1: Ten most significant up-regulated genes of U87 in 3D vs. 2D using RNA-seq, Table S2: Ten most significant down-regulated genes of U87 in 3D vs. 2D using RNA-seq, Video S1: U87-td-farnesyl expressing cells in 3D Matrigel without scaffold, Video S2: U87-td-farnesyl in Matrigel with in 3D Matrigel with scaffold, Video S3: U87 on aligned 3D microfibers, Video S4: LN18 on aligned 3D microfibers, Video S5: LN18 in the presence of PTEN inhibitor on 3D aligned microfibers, Video S6: MDA-MB-468 on aligned 3D microfibers, Video S7: MDA-MB-231 on aligned 3D microfibers, Video S8: U87 in the presence of RHO inhibitor on 3D aligned microfiber, Video S9: U87 skating snail on 3D aligned microfibers, Video S10: MDA-MB-468 skating snail on 3D aligned microfibers.
