# Supplementary material for: Brain and Breast Cancer Cells with PTEN Loss of Function Reveal Enhanced Durotaxis and RHOB Dependent Amoeboid Migration Utilizing 3D Scaffolds and Aligned Microfiber Tracts
Source: Cancers (Basel). 2021 Oct 14;13(20):5144. doi: 10.3390/cancers13205144 (PMC8533830; doi:10.3390/cancers13205144)
Supplement: Supplementary file 1 [file cancers-13-05144-s001.zip › cancers-1376978-supplementary/cancers-1376978 supplementary after proof.pdf]

cancers MDPI

Supplementary Material: Brain and Breast Cancer Cells with PTEN Loss of Function Reveal Enhanced Durotaxis and RHOB Dependent Amoeboid Migration utilizing 3D Scaffolds and Aligned Microfiber Tracts

Annalena Wieland, Pamela L. Strissel, Hannah Schorle, Ezgi Bakirci, Dieter Janzen, Matthias W. Beckmann, Markus Eckstein, Paul D. Dalton and Reiner Strick

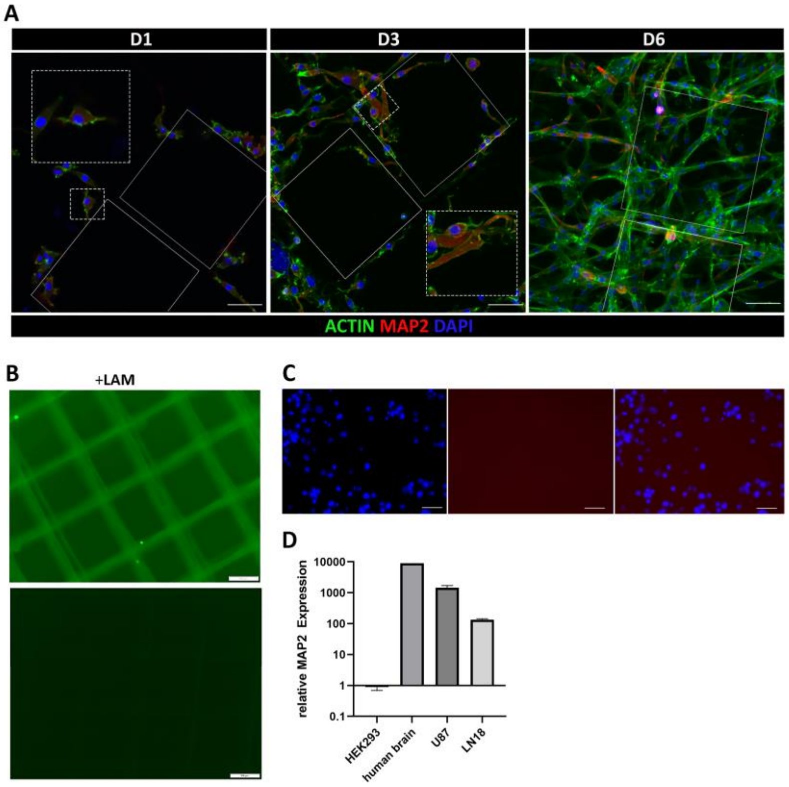

Figure S1. Durotaxis, topotaxis and cell adhesion / network formation on 3D scaffolds: **(A)** Representative confocal image z-stacks of U87 GBM PTEN loss of function cells grown in 3D with Matrigel (4.5 mg mL^{-1}) and scaffolds in a time kinetic manner at day 1, 3 and 6. Note cell attraction of amoeboid cells (D1, D3) and cell networks (D6) on scaffolds (white solid squares indicate a single box pore (200 µm); small single dotted white squares show specific amoeboid cells with prominent F-actin staining of cellular protrusions and around the cell circumference and nuclei are located towards the cell rear; large dotted white squares show a magnification of amoeboid cells represented from the small dotted white squares (green = F-Actin Alexa 488, red = MAP2, blue = nuclei) (scale bar = 30 µm). (**B)** Detection of laminin-111 (LAM) coating of a scaffold using a specific laminin antibody (green) (top), 2nd antibody as a negative control (below) (scale bar = 100 µm). (**C)** From left to right: U87 cells seeded on cover slips and nuclei stained with DAPI; hybridized with secondary antibody goat anti mouse Dylight 594 alone and a merged image of DAPI and secondary antibody. Bar = 50 µm. **(D)** qPCR of HEK293, purchased human brain, U87 and LN18 using specific primers for MAP2. Y-axes show relative expression of MAP2.

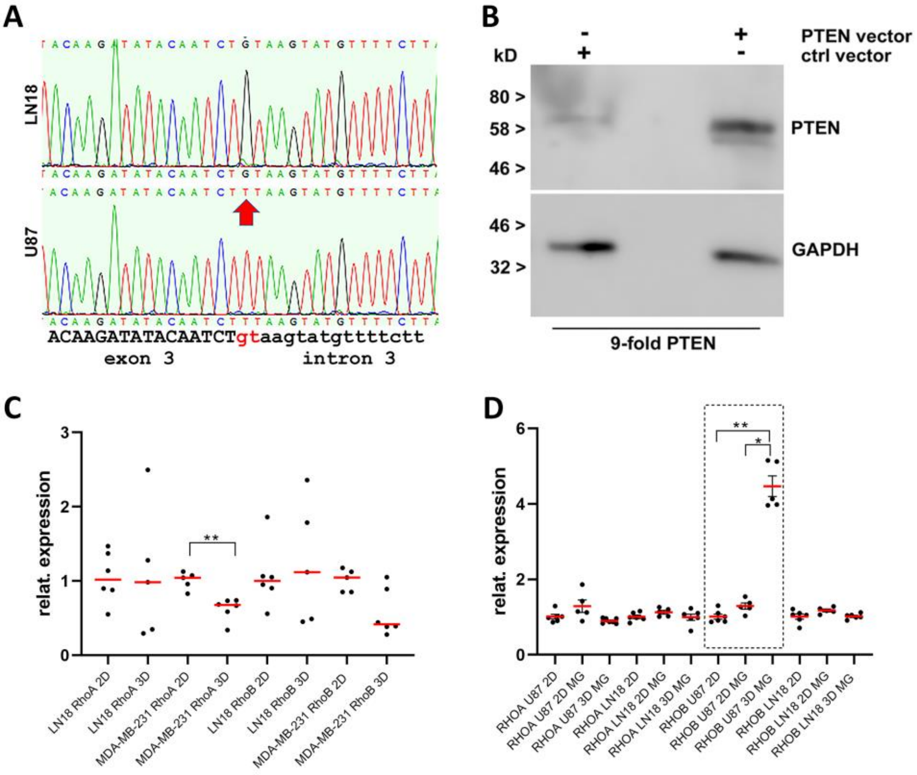

Figure S2. PTEN splice mutation analysis and RHOA / RHOB expression of GBM and TNBC cells: (**A)** DNA from LN18 GBM *PTEN* wt cells (top) and U87 GBM PTEN loss of function cells (bottom) was sequenced for *PTEN* exon 2 and intron 3. Splice donor site of exon 3 (GT) is mutated to TT in U87 GBM cells (red arrow). (**B)** Western Blot showing PTEN protein expression at 24 h after transfection with control vector (pcDNA4/myc-HisA) (left). Left lane represents U87 GBM cell line with homozygote PTEN loss of protein expression and function. Right lanes represent *PTEN* transfection and rescue with 9-fold higher PTEN expression levels (normalized to GAPDH, lower bands) after 24 h (right lane) compared to cells transfected with control vector (left lane). (**C)** qPCR and relative gene expression (2-DDCt) of *RHOA* and *RHOB* in LN18 GBM and MDA-MB-231 cells grown for 3 days in 3D with Matrigel and scaffolds compared with 2D; ** $p = 0.0043$. (**D)** qPCR and relative gene expression (2-DDCt) of *RHOA* and *RHOB* in U87 and LN18 cells grown for 3 days in 2D, 2D with Matrigel (2D MG) coating and 3D with Matrigel (3D MG) without scaffolds; * $p = 0.0079$, ** $p = 0.0043$.

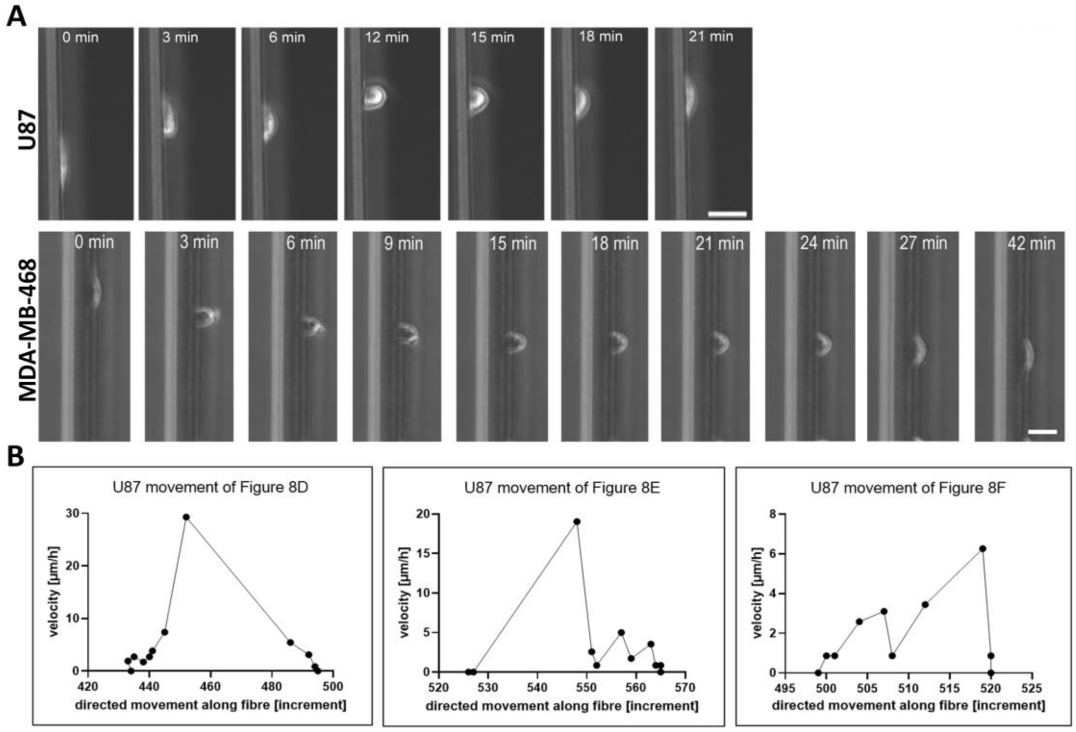

Figure S3. The skating snail-like cellular plasticity modes of migration. **A)** Representative images of U87 GBM PTEN loss of function (top) and MDA-MB-468 PTEN loss of function (bottom) cell migration over time (min) (scale bar = 25 µm). Note the similar cellular plasticity of U87 GBM and MDA-MB-468 cells beginning from a non-moving cell with a flat morphology to a round amoeboid cell shape: for U87 GBM cells images represent from 12–15 min and for MDA-MB-468 cells from 3–21 min. **B)** Graphs showing velocity in µm/h (Y = axis) of U87 GBM cell movements along aligned microfibers (X-axis in increments) correlating with cellular plasticity modes in Figure 8A, D (left graph); Figure 8E (middle graph) and Figure 8B, F (right graph).

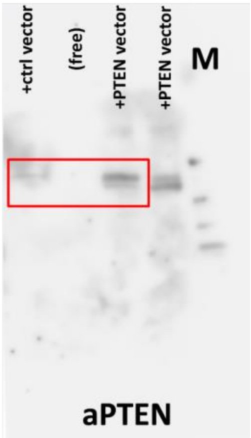

Figure S4: An immunoblot of U87 cell lysates after transfection with PTEN (PTEN vector) and control (vector) hybridized with an antibody specific for PTEN (aPTEN) is shown. This is the uncropped immunoblot for Figure S2B (upper). M = protein marker.

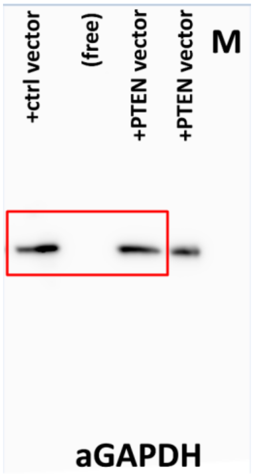

Figure S5: Immunoblot Figure S4 hybridized with an antibody for GAPDH protein (aGAPDH). This is the uncropped immunoblot for Figure S2B (down). M = protein marker.
